# The Role of Intergranular Cracks on Fast Charging and Accelerated Degradation in Polycrystalline Layered Oxide Cathodes

**DOI:** 10.1002/advs.202515588

**Published:** 2025-10-29

**Authors:** Jinhong Min, Tobias Glossmann, Yiyang Li

**Affiliations:** ^1^ Materials Science and Engineering University of Michigan Ann Arbor Michigan 48109 USA; ^2^ Mercedes‐Benz Research & Development North America Farmington Hills MI 48331 USA

**Keywords:** intergranular cracks, lithium‐ion batteries, NMC cathodes, polycrystalline particles, single‐crystalline particles

## Abstract

Polycrystalline layered oxide cathodes such as Li(Ni_x_Mn_y_Co_z_)O_2_ (NMC) and Li(Ni_x_Co_y_Al_z_)O_2_ (NCA) are some of the most widely used materials for Li‐ion batteries. Due to anisotropic volume expansion upon lithium insertion and removal, such particles undergo substantial intergranular cracks. In this article, we presents a perspective on both the positive and negative roles of cracks on the electrochemical performance of these polycrystalline cathode particles based on recent research. Like in many other systems, cracks and fractures result in performance degradation and failure. However, in layered oxide particles, they also play a critical role in faster reaction kinetics by enabling electrolyte penetration, thereby shortening the diffusion length and increasing the reaction area. Acknowledging this dual nature of cracks and electrolyte penetration is critical in understanding how cathode materials are analyzed, modeled, and engineered for future energy storage applications.

## Graphical abstract

Polycrystalline layered oxide cathodes, such as Li(Ni,Mn,Co)O_2_, create intergranular cracks from anisotropic volume changes during electrochemical cycling. These cracks negatively affect structural integrity and accelerate particle degradation, but also increase the surface area and thereby enable fast charge and discharge. Acknowledging this dual role is crucial for analyzing, modeling, and engineering cathode materials for advanced energy storage.

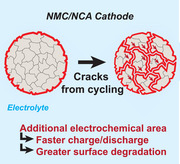

## 1 Introduction

Layered lithium metal oxides based on LiMO_2_ (M = Ni, Mn, Co, Al) offer cathode materials with the highest energy densities for Li‐ion batteries, and are widely used in electric vehicles. The most common morphology utilizes spherical secondary particles (5–50 µm) made up polycrystalline agglomerate of primary particles (100–1000 nm), sometimes referred to as “meatballs.”^[^ ^1^, ^2^, ^3^ ^]^ Due to anisotropic volume expansion upon the insertion and removal of lithium,^[^ ^4^, ^5^, ^6^ ^]^ these particles can crack and fracture along grain boundaries during electrochemical cycling. Understanding the role of cracks on electrochemical properties is critically important to battery science and engineering.

It is long recognized that intergranular cracks are a major cause of capacity degradation during cycling.^[^ ^7^, ^8^, ^9^, ^10^, ^11^, ^12^, ^13^, ^14^ ^]^ These cracks promote electrolyte infiltration, accelerating cycle life degradation as the surfaces of the particles react with the electrolyte. The degradation mechanisms at the interface between the particle surface and the electrolyte have been extensively investigated and reviewed.^[^ ^15^, ^16^, ^17^ ^]^ In particular, Ni‐rich compositions, which are widely studied to meet growing demands for high energy density, are known to exhibit more severe cracking and faster capacity fade under high‐voltage charging conditions.^[^ ^18^, ^19^, ^20^, ^21^, ^22^, ^23^ ^]^ To address these issues, strategies such as surface coatings to protect the reactive interface from the electrolyte,^[^ ^24^ ^]^ doping to enhance surface stability,^[^ ^25^ ^]^ and microstructural engineering approaches^[^ ^26^ ^]^ have been proposed. Among them, the synthesis of single‐crystal particles, which eliminates intergranular cracks between primary particles, has attracted significant attention.^[^ ^27^, ^28^, ^29^, ^30^, ^31^, ^32^, ^33^, ^34^ ^]^

While the role of cracks in mechanical degradation is not disputed, most research papers have not considered their critical role in enabling fast charge and discharge. Recently, several studies have shown clear and compelling evidence that the electrolyte penetration into intergranular cracks increases the electrochemically active surface area and reduces the diffusion length.^[^ ^35^, ^36^, ^37^, ^38^, ^39^ ^]^ However, these relatively recent observations have not been integrated into a perspective or review paper that simultaneously recognizes the dual role of cracks in layered oxide cathodes. Instead, most review and perspective papers only highlight cracks as sources of degradation.^[^ ^15^, ^16^, ^18^, ^31^, ^33^, ^34^, ^40^, ^41^, ^42^, ^43^ ^]^ Without considering their positive effects on electrochemical kinetics, efforts to eliminate cracks could lead to unintentional consequences.

In this Perspective, we systemically review both the positive and negative roles of intergranular cracks in layered oxide cathodes, primarily Li(Ni_x_Mn_y_Co_z_)O_2_ (NMC) and Li(Ni_x_Co_y_Al_z_)O_2_ (NCA) (x+y+z = 1). We first review the synthesis routes that lead to polycrystalline morphologies and the structural origins of intergranular cracks. Next, we review the relationship between cracking and capacity degradation as well as its role in controlling electrochemical kinetics. Finally, we discuss future directions for cathode design and modeling strategies that explicitly incorporate the dual nature of intergranular cracking.

## 2 Synthesis and Microstructure of Layered Oxide Cathode Particles

**Figure 1:**
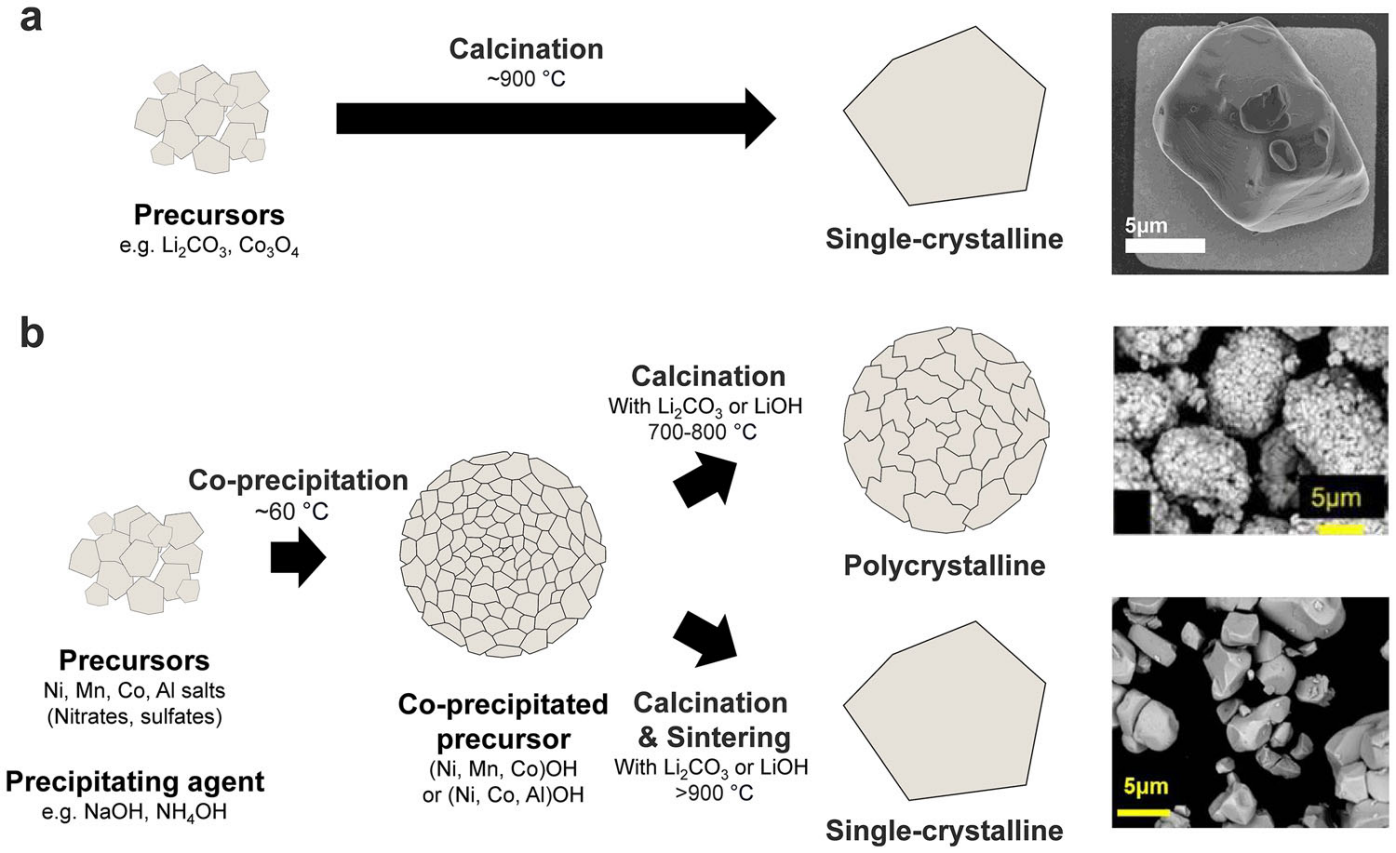
Synthesis of layered oxide cathode particles. a) Schematic illustration of solid‐state synthesis and an example of synthesized, micron‐sized LiCoO_2_ particles.^[^ ^50^ ^]^ In this method, precursors are thoroughly mixed and calcined at high temperatures. Image reproduced with permission from ref. [50]. Copyright 2025, American Chemical Society. b) Schematic illustration of co‐precipitation synthesis for NMC and NCA cathodes. Transition metal hydroxide precursors are formed at low temperatures and subsequently calcined with lithium salts to yield polycrystalline cathode particles. During high‐temperature sintering, the polycrystalline morphology transforms into a single‐crystalline structure.^[^ ^27^ ^]^ Image reproduced with permission from ref. [27]. Copyright 2024, IOP Publishing.

During the early development of lithium‐ion batteries, solid‐state synthesis was the most widely adopted route for synthesizing oxide‐based cathode materials,^[^ ^44^ ^]^ including for the first oxide cathode LiCoO_2_ (LCO) by the group of John Goodenough in 1984.^[^ ^45^ ^]^ By mixing the precursors and calcining at temperatures above 900 °C,^[^ ^29^, ^30^, ^45^ ^]^ most solid‐state syntheses yielded single‐crystal particles on the order of 10 µm (**Figure** **1**a). However, solid‐state synthesis presents several limitations. To ensure sufficient diffusion of the transition metal ions in the structure, the synthesis required prolonged calcination at high temperatures, often several days at 900 °C or higher.^[^ ^46^ ^]^ Additionally, Ostwald ripening during high‐temperature treatment often caused excessive particle growth.^[^ ^47^ ^]^ Such particles were effective for early cathode materials such as LiCoO_2_ and the spinel LiMn_2_O_4_ with a single transition metal.

While reducing the calcination temperature can reduce energy consumption and yield smaller particles, insufficient diffusion of transition metals at lower temperatures may prevent the formation of the correct phase and composition.^[^ ^48^, ^49^ ^]^ This challenge is especially acute for NMC and NCA, which contain at least three different transition metals that should be uniformly mixed in the solid. Although LiCoO_2_ is still used in portable electronics with smaller battery packs, NMC and NCA cathodes are preferred in electric vehicles and grid storage due to the lower cost of Ni and Mn compared to Co.

Co‐precipitation synthesis provided an avenue to overcome these challenges^[^ ^51^ ^]^ and was popularized in the early 2000s. Co‐precipitation involves two synthetic steps. The first step creates the transition metal hydroxide precursors in aqueous solution at low temperatures (≈60 °C) with the correct ratio and distribution of transition metals. The second step calcines this hydroxide precursor with lithium salts at 700–800 °C to form the final NMC/NCA cathode material. Co‐precipitation synthesis combines fast transition metal diffusion in the liquid phase to yield uniform transition metal mixtures with the much faster lithium ion diffusion in the solid during calcination. Compared to the solid‐state synthesis, co‐precipitation enables shorter calcination durations and lower processing temperatures while still reliably forming the layered oxide structures.^[^ ^52^ ^]^ More detailed information on the synthesis of cathode particles for lithium‐ion batteries via co‐precipitation can be found in the review article by Dong and Koenig.^[^ ^53^ ^]^

The hydroxide precursors typically consist of agglomerated primary particles that form secondary spherical particles, a morphology that is retained after calcination (Figure 1b). As a result, the final cathodes exhibit a polycrystalline structure composed of 100–1000 nm primary particles assembled into large 5–20 µm secondary particles, sometimes referred to as a “meatball.” The size of the primary particles can be tuned by adjusting the calcination temperature and time, while the size of the secondary particles can be tuned through tuning parameters in the low‐temperature co‐precipitation steps. More details about the process to control the size, shape, and morphology can be found in a review article by Malik and colleagues.^[^ ^54^ ^]^

In the past decade, several groups have utilized co‐precipitation to synthesize single‐crystal NMC (Figure 1b).^[^ ^8^, ^27^, ^55^, ^56^, ^57^, ^58^, ^59^, ^60^ ^]^ These single crystals are synthesized by applying excess Li and higher calcination temperatures, often above 900 °C. Alternative methods to form single‐crystal NMC include molten salt synthesis^[^ ^29^, ^61^ ^]^ but are less scalable for the large batches expected commercially. More detailed discussion on the synthesis of single‐crystalline cathode particles for lithium‐ion batteries can be found in the review article by Langdon and Manthiram.^[^ ^31^ ^]^

While there exists interest in single‐crystal particles, most commercial NMC and NCA cathodes utilize a polycrystalline structure due to the scalability, flexibility, and lower temperatures of co‐precipitation synthesis. This polycrystalline morphology arises from the agglomeration of sub‐micron primary particles into micrometer‐scale secondary particles during synthesis and calcination.

## 3 Intergranular Cracks and Performance Degradation in Polycrystalline Particles

**Figure 2:**
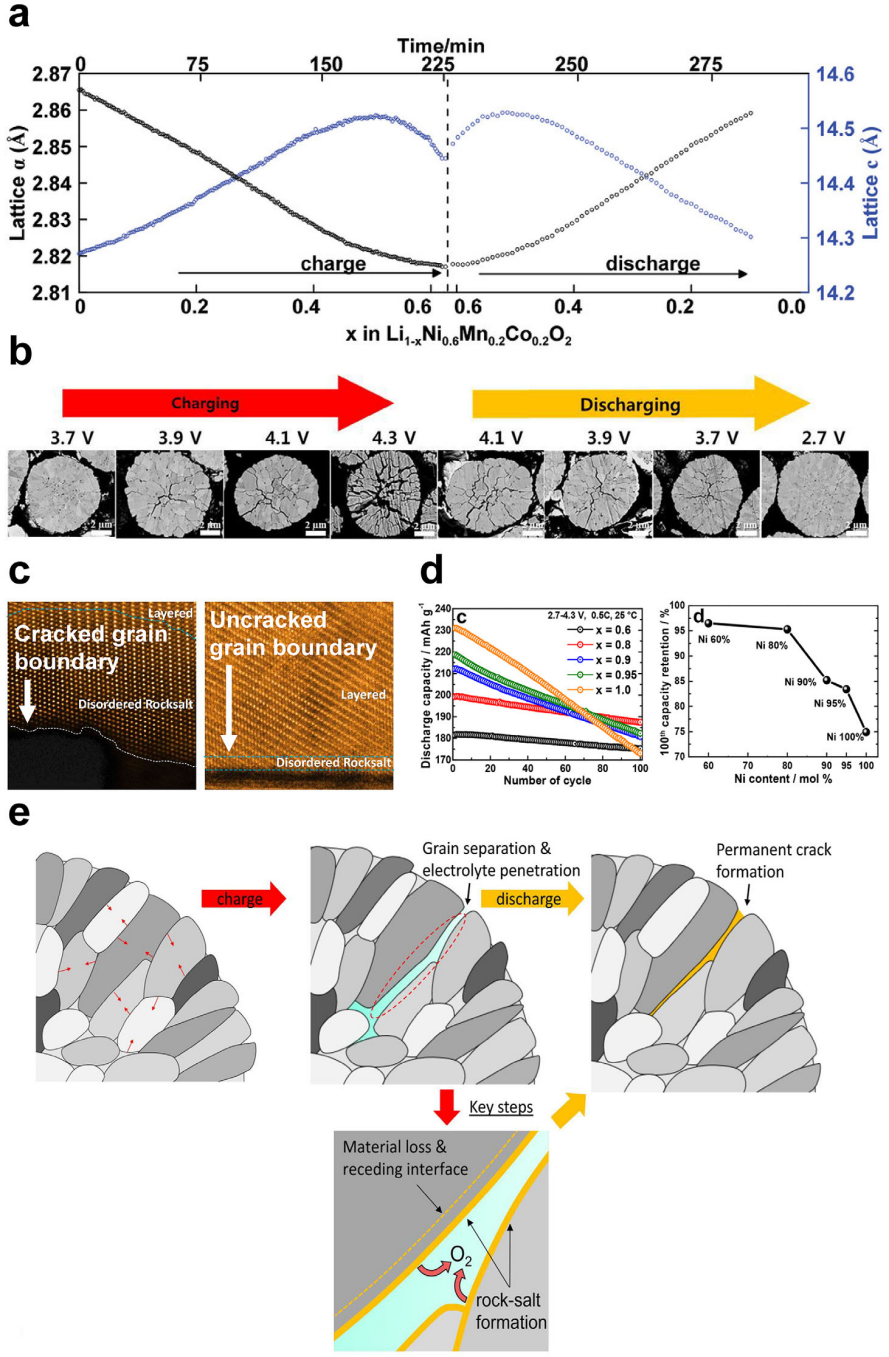
Degradation of NMC/NCA cathodes from cracking and electrolyte penetration. a) In situ X‐ray diffraction shows lattice expansion and contraction during electrochemical charge and discharge. Image reproduced with permission from ref. [103]. Copyright 2014, Wiley. b) Cross‐sectional images of polycrystalline NCA cathodes charged to different cutoff voltages during the first charge and discharge show intergranular cracking, which partially closes during discharge. Image reproduced with permission from ref. [20]. Copyright 2019, American Chemical Society. c) High‐resolution transmission electron microscopy shows that cracked boundaries lead to surface reconstruction, a signature of interfacial reactions with the electrolyte, which has penetrated the cracks. In contrast, sealed grain boundaries without cracks show no signs of degradation. Image reproduced with permission from ref. [77]. Copyright 2020, American Chemical Society. d) When cycled to the same cutoff voltage, Ni‐rich NMC particles exhibit more rapid capacity fading compared to lower‐Ni compositions. Image reproduced with permission from ref. [21]. Copyright 2018, American Chemical Society. e) Lee, Manthiram, and coworkers’ summary schematic of NMC degradation as a result of cracks and electrolyte penetration. The infiltrated electrolyte reacts with the particle surface, promoting surface degradation and further crack propagation. Image reproduced with permission from ref. [18]. Copyright 2023, Elsevier.

Layered cathode materials utilize the intercalation and deintercalation of lithium ions during charge and discharge; this change in lithium content and transition metal valence yields chemical expansion and contraction ^[^ ^4^, ^6^, ^7^, ^10^, ^20^, ^23^, ^62^, ^63^, ^64^, ^65^, ^66^, ^67^, ^68^ ^]^ (**Figure** **2**a). As lithium is extracted, the oxidation state of nickel typically increases from Ni^2+^ to Ni^3+^ or Ni^4+^, shortening the TM–O bond lengths and causing contraction along the *a*‐axis. The *c*‐axis exhibits a non‐monotonic behavior, expanding during initial delithiation and contracting at later stages, due to changes in electrostatic repulsion between transition metal slabs. Such lattice contraction becomes more pronounced at higher potentials and in compositions with higher Ni content under the same potential. According to Kondrakov and colleagues, when charged to 4.3 V, NMC111 (low Ni) exhibits a volume contraction of ≈1.2%, whereas NMC811 (Ni‐rich) shows ≈5% contraction^[^ ^3^ ^]^ at the same potential. The volume expansion and contraction of individual particles were also directly visualized in real space using operando scanning electron microscopy.^[^ ^69^ ^]^

Lattice contraction and expansion apply mechanical stress to the particles, which induces the formation and propagation of cracks.^[^ ^9^, ^22^, ^70^, ^71^ ^]^ Cracks can be classified into two types: intragranular cracks and intergranular cracks. Intragranular cracks occur parallel to the (003) plane of the lattice; after repeated charge and discharge cycles, they develop and eventually penetrate through the primary particles. Yan and colleagues observed the formation of intergranular cracks in NMC111 particles depending on the cutoff voltage.^[^ ^71^ ^]^ Such cracks initiate from dislocations inside the particles, where mechanical stress caused by lithium concentration gradients is concentrated, and these stresses evolve into cracks. Lin and colleagues observed that intragranular cracks originate from regions where Li‐Ni antisite defects are aggregated in NMC811.^[^ ^9^ ^]^ Bi and colleagues observed that in NMC76 particles, the (003) planes can reversibly glide and return during cycling, but dislocations accumulate that can eventually evolve into intragranular cracks.^[^ ^72^ ^]^

Mechanical stress caused by lattice contraction and expansion also induces intergranular cracks. NMC and NCA materials are usually synthesized by co‐precipitation, forming polycrystalline secondary particles composed of many primary particles (Figure 1b). Prior to electrochemical cycling, these primary particles are tightly packed with dense grain boundaries. Because these grains are oriented in different directions, anisotropic lattice contraction during lithium removal in the first cycle induces substantial stress at the grain boundaries of adjacent particles.^[^ ^21^, ^67^, ^71^, ^73^ ^]^ When such stresses exceed the cohesive force at grain boundaries, intergranular cracks would initiate.^[^ ^21^, ^71^, ^74^, ^75^ ^]^ Upon further lithium removal and lattice expansion and contraction, the additional stress would build that causes the intergranular cracks to propagate along the grain boundaries. This effect becomes more severe as delithiation progresses or when a sharp change in lattice parameters occurs during the H2–H3 phase transition in Ni‐rich compositions.^[^ ^20^, ^22^, ^62^, ^68^ ^]^ Although such cracks form during lithium removal (charging), they can close during lithium insertion (discharge) as the particles expand. Nam and colleagues visualized the formation of cracks during the first charge in polycrystalline Ni‐rich NCA particles (Figure 2b),^[^ ^20^ ^]^ and showed that lattice contraction and expansion can open and close cracks.

Liquid electrolytes can penetrate the particles’ interior through intergranular cracks induced by mechanical stress. Electrolyte penetration increases the electrochemical reaction surface area and accelerates degradation. There are at least three related degradation mechanisms associated with higher voltages and higher states of charge, or lower lithium concentration. First, when the cathode is charged to high voltage, nickel at the surface is reduced while releasing oxygen. As a result, the layered structure is irreversibly reconstructed into a rock salt structure.^[^ ^68^, ^76^, ^77^ ^]^ Since the rock salt structure has a significantly lower lithium diffusion rate, it increases impedance and negatively affects both the capacity as well as the reaction kinetics of the cathode material.^[^ ^68^, ^76^, ^78^, ^79^, ^80^ ^]^ In nickel‐rich NMC particles, this reconstruction into the rock salt structure occurs even at lower voltages.^[^ ^81^ ^]^ This surface reconstruction from layered to rock salt can also be a source of additional stress and crack propagation over extended cycling.^[^ ^73^ ^]^ Second, the transition metals can dissolve over time upon extended cycling,^[^ ^82^, ^83^, ^84^, ^85^ ^]^ resulting in a loss of material and making cracks appear larger. This transition metal dissolution is partly a result of the formation of HF from the reaction of the LiPF_6_ salt with residual water in the electrolyte.^[^ ^86^, ^87^, ^88^ ^]^ Transition metal dissolution in layered cathodes is not as severe as in Mn‐rich cathodes like the spinel LiMn_2_O_4_.^[^ ^89^ ^]^ In Ni‐rich compounds like NMC811, Ni dissolution becomes more significant at high potentials and extended cycling.^[^ ^90^, ^91^ ^]^ Third, at high voltage, the surface reactivity of NMC particles increases, causing electrolyte decomposition and the formation of a resistive cathode electrolyte interphase layer (CEI).^[^ ^13^, ^92^, ^93^, ^94^ ^]^ The CEI thickness was found to vary with the Ni content of NMC cathode material, suggesting differences in reactivity of the electrolyte with the cathode material.^[^ ^90^ ^]^ Takahashi et al. made a similar discovery, comparing Ni‐rich with Li‐rich NMC materials.^[^ ^95^ ^]^ High cell voltage (e.g., 4.6 V) may also lead to the formation of acidic electrolyte oxidation products that may thin the CEI.^[^ ^96^ ^]^ West et al. discovered a more organic‐rich CEI with lower relative amounts of LiF because of higher voltage (4.3 vs 4.1 V) cycling of NMC811.^[^ ^90^ ^]^

Intergranular cracking exacerbates these degradation processes because the electrolyte would penetrate the polycrystalline secondary particles.^[^ ^97^, ^98^ ^]^ This electrolyte penetration expands the electrochemically active surface to within the bulk of the particle, resulting in internal surface degradation. Zou and colleagues studied polycrystalline LiNi_0.76_Mn_0.14_Co_0.10_O_2_ after 200 cycles using scanning transmission electron microscopy. They found that rock‐salt layers, ≈5 nm thick, formed along cracked internal grain boundaries but not along sealed, intact ones (Figure 2c).^[^ ^77^ ^]^ These results suggest that cracks facilitate electrolyte infiltration, expanding the electrochemically active surface area and promoting degradation through the processes described earlier. Recent research by Morzy and colleagues showed that the electrolyte can also penetrate into intragranular cracks upon extended cycling in Ni‐rich cathodes;^[^ ^99^ ^]^ however, there are considerably fewer intragranular cracks with electrolyte penetration compared to intergranular cracks. We focus on intergranular cracks in this perspective article, because electrolyte penetration is responsible for enhanced electrochemical kinetics and most of the degradation.

As cycling continues, surface degradation along the cracks becomes more severe, and the associated material loss leads to larger cracks, making them increasingly visible under electron and even X‐ray microscopy.^[^ ^9^, ^10^, ^19^, ^100^, ^101^ ^]^ This degradation is accompanied by a decline in the reversible capacity and an increase in the impedance.^[^ ^68^, ^76^, ^102^ ^]^ Such crack evolution and capacity fading are particularly prominent at high voltages and in Ni‐rich compositions. While Ni‐rich NMCs offer high capacity, they are prone to more severe cracking due to sharp lattice changes during the H2–H3 phase transition. Repeated cycling accelerates the surface degradation along the cracks and leads to rapid capacity loss. Ryu and colleagues synthesized NMC particles with nickel content ranging from 0.6 to 0.95 and quantified the crack formation, lattice parameter changes, and discharge capacity loss.^[^ ^21^ ^]^ They confirmed that higher nickel content leads to more extensive cracking and faster capacity fading (Figure 2d). These observations have drawn significant attention to the relationship between crack formation and degradation, with many interpreting the increased electrochemical surface area caused by cracking as a driver of degradation. These conclusions were summarized in a recent perspective paper by Lee and colleagues (Figure 2e).^[^ ^18^ ^]^

## 4 Enhanced Reaction Kinetics from Intergranular Cracking

The previous section discussed the degradation caused by electrolyte infiltration upon cracks. Based on this assessment, it is believed that reducing or eliminating cracks would improve cycle life. For this reason, many researchers sought to eliminate intergranular cracking, including by using single‐crystal particles that do not have sintered grain boundaries.^[^ ^22^, ^28^, ^31^, ^104^, ^105^ ^]^ Other methods to reduce cracks include better alignment of the primary particles to alleviate internal stress.^[^ ^106^, ^107^ ^]^ However, recent research has shown that this electrolyte penetration also enables faster electrochemical kinetics and rate capability. While substantial progress has been made in understanding the relationship between cracking and degradation in efforts to improve NMC battery longevity, the kinetic benefits of cracks, particularly the increased electrochemical surface area and enhanced (de)lithiation rates, have received comparatively less attention.^[^ ^22^, ^28^, ^31^, ^104^, ^105^ ^]^

While cracks and surface damage have primarily been characterized and visualized using microscopy (Figure 2), changes in electrochemical rate kinetics are typically characterized using electrochemical techniques, particularly Potentiostatic Intermittent Titration Technique ^[^ ^37^, ^108^ ^]^ (PITT), Galvanostatic Intermittent Titration Technique ^[^ ^35^, ^109^, ^110^, ^111^ ^]^ (GITT), and Electrochemical Impedance Spectroscopy^[^ ^82^, ^112^ ^]^ (EIS). These small‐signal techniques are performed near the open‐circuit voltage and rely on current, voltage, and time responses to extract parameters such as exchange current density and solid‐state lithium diffusion coefficients.^[^ ^113^ ^]^

One challenge is that electrochemical measurements provide the resistance or time constants. To obtain material parameters like the lithium diffusion coefficients or the exchange current density, the surface area and diffusion length must be inputted as parameters. In the early days of Li‐ion battery development, most cathodes consisted of micron‐sized, single‐crystal particles such as LiCoO_2_^[^ ^114^, ^115^, ^116^ ^]^ or LiMn_2_O_4_.^[^ ^117^, ^118^, ^119^ ^]^ Here, diffusion lengths and surface areas could be estimated from the particle size distribution. However, electrochemical techniques cannot easily detect the change in electrochemically active surface area that results from crack formation during (de)lithiation in polycrystalline particles. As a result, it is difficult to distinguish whether the observed changes in the reaction or diffusion coefficients arise from intrinsic changes in the material properties during lithium insertion and removal, or from the changes to the geometry due to cracking.

**Figure 3:**
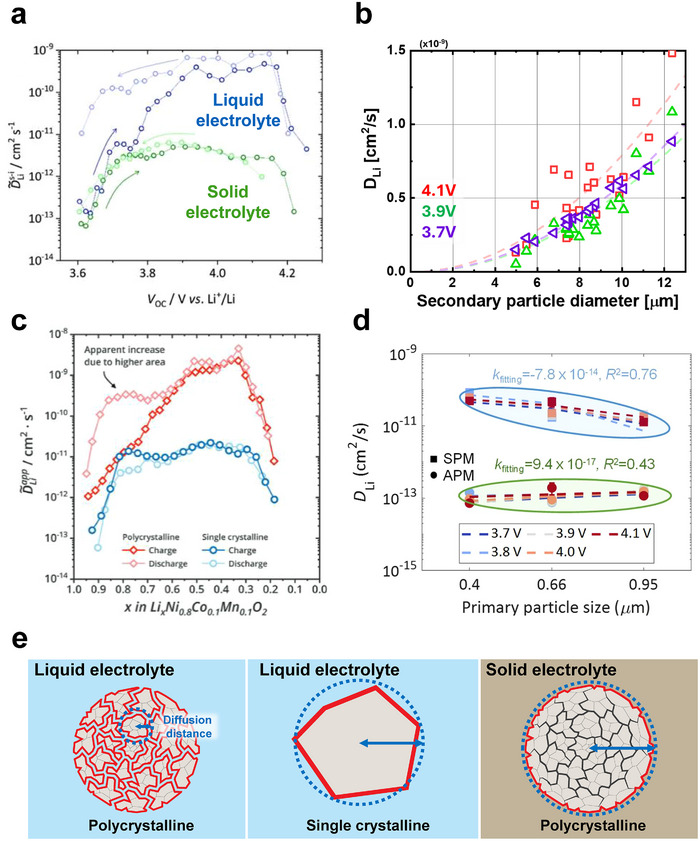
Effects of electrolyte penetration on the rate kinetics of NMC cathode particles. a) NMC particles in liquid electrolytes exhibit an apparent two‐order‐of‐magnitude increase in the lithium diffusion coefficient compared to solid electrolytes.^[^ ^35^ ^]^ At low states of charge and open‐circuit voltages, the liquid electrolyte also exhibits hysteresis between first charge and discharge. This result is attributed to cracks and electrolyte penetration during the first cycle in the liquid but not the solid electrolyte. Image reproduced with permission from ref. [35]. Copyright 2020, IOP Publishing. b) Single particle electrochemistry shows that the apparent diffusion coefficient increases with the size of polycrystalline secondary particles in a monolithic solid particle model. Image adapted with permission from ref. [37]. Copyright 2023, Royal Society of Chemistry. c) A comparison of single‐crystal and polycrystalline particles shows that the lithium diffusivity appears to be much higher in polycrystalline than in single‐crystalline particles. This result is attributed to cracks that form in polycrystalline rather than single‐crystal particles. Image reproduced with permission from ref. [36]. Copyright 2021, Wiley. d) Diffusion coefficient estimated with monolithic solid particle model (SPM) depends on the primary particle size, but the model that considers the electrolyte penetration and the size of the primary particle (APM) shows a constant diffusion coefficient regardless of the primary particle size. Image reproduced with permission from ref. [120] Copyright 2025, Elsevier. e) Schematics of electrolyte penetration in polycrystalline and single‐crystalline particles for both liquid and solid electrolytes. In polycrystalline particles, liquid electrolytes can infiltrate along intergranular cracks, shortening the diffusion length. In contrast, single‐crystal particles do not have intergranular cracks, while solid electrolytes cannot penetrate the cracked grains of polycrystalline particles.

Several recent studies have employed carefully designed experiments to directly observe changes in reaction and diffusion kinetics induced by cracks formed during the first charge. Ruess and colleagues measured the apparent diffusion coefficients of polycrystalline Ni‐rich NMC811 in both solid and liquid electrolytes using galvanostatic intermittent titration^[^ ^35^ ^]^ under the assumption that the diffusion length is the size of the secondary particle. In a liquid electrolyte, the lithium diffusion coefficient is initially low; however, upon delithiation, the apparent diffusion coefficient rapidly increases. Importantly, after fully discharging the cathode, the apparent diffusion coefficient is much higher than the initial, pristine state (**Figure** **3**a). This result was interpreted as the liquid electrolyte penetrating the intergranular cracks such that the diffusion length is substantially decreased; this will give the appearance of an increase in lithium diffusivity. In the same paper, this effect was not observed when using solid electrolytes,^[^ ^35^ ^]^ which are assumed not to be able to flow into the cracks.

The kinetic benefits arising from crack formation during the first charge cycle were also observed by Min and colleagues through single‐particle electrochemical analysis. This study compared the lithium reaction and diffusion coefficients of 21 NMC532 particles of different particle sizes using PITT.^[^ ^37^ ^]^ Their study observed that when polycrystalline particles were modeled as monolithic solids, the diffusion coefficients increased with larger particles (Figure 3b). This result suggests that the monolithic solid particle model did not account for the penetration of liquid electrolyte through the cracks, leading to an increase in the apparent diffusion coefficient. This result was also observed by Zuo and colleagues with single‐particle electrochemistry in Ni‐rich NMC.^[^ ^120^ ^]^

The influence of cracking on electrochemical kinetics is further highlighted in direct comparisons between single‐crystalline and polycrystalline particles. On the porous electrode level, Trevisanello and colleagues compared the apparent diffusion coefficients of single‐ and poly‐crystalline NMC^[^ ^36^ ^]^ in liquid electrolytes. They measured the evolution of diffusion coefficients during the first charge and discharge cycles of Ni‐rich NMC811 using EIS in liquid electrolytes. In single‐crystalline particles, the diffusion coefficients during charge and discharge were nearly identical (Figure 3c), similar to that of the solid electrolyte previously discussed (Figure 3a). However, in polycrystalline NMC, the diffusion coefficient increased significantly during discharge compared to charge (Figure 3c). This behavior can be attributed to crack formation in polycrystalline particles, which increases the electrochemical surface area and thus the apparent diffusion coefficient after the first charge. In contrast, single‐crystalline NMC lacks internal grain boundaries or intergranular cracks, so its electrochemical surface area does not change substantially, resulting in consistent diffusion behavior across cycles.^[^ ^36^ ^]^ Min and colleagues also showed that single‐crystal NMC particles show substantially lower rate capability using single‐particle electrochemical measurements.^[^ ^38^ ^]^

Zuo and colleagues evaluated the lithium diffusion coefficient of polycrystalline Ni‐rich NMC composed of primary particles with various sizes using single particle electrochemistry.^[^ ^120^ ^]^ They observed that under the assumption of no electrolyte penetration, the diffusion coefficient appeared to decrease with larger primary particles. Instead, if the characteristic diffusion length is given by the diameter of the primary particles, the diffusion coefficient remained constant regardless of the primary particle size (Figure 3d). This not only confirms electrolyte penetration into the secondary particles but also implies that the rate capability of cathode particles can be tuned by controlling the size of the primary particles.

Figure 3e shows our interpretation of the conclusions from these results. In polycrystalline particles in liquid electrolytes, electrolyte penetration enables substantially shorter lithium diffusion lengths. In single‐crystal particles in liquid electrolytes, the absence of electrolyte penetration results in substantially longer lithium diffusion lengths. Finally, in polycrystalline particles in solid electrolytes, the electrolyte also does not penetrate the secondary particle. The same effect is expected in single‐crystal particles in solid electrolytes. Presently, it is not resolved whether the relevant diffusion length is a single primary particle or several primary particles, and is subject to future investigation.

One question is whether cracks or porous structures could be deliberately engineered. Indeed, several groups have specifically synthesized nanoporous NMC structures^[^ ^121^, ^122^, ^123^ ^]^ with the goal of reducing cracking and improving lithium diffusion. However, we believe that porous structures have several shortcomings compared to intergranular cracks that form during cycling. First, pores replace active material with free space, thereby reducing the volumetric energy density of the cell. At tens of nanometers, engineered pores are larger than the cracks that form upon initial cycling. Second, deliberately engineering the desired pores would usually require additional or more complex processing. Finally, such nanoporous structures could accelerate particle degradation prior to cycling, both during storage and during fabrication. It is well documented that Ni‐rich NMCs degrade when stored in ambient air;^[^ ^124^, ^125^, ^126^, ^127^ ^]^ although they are more stable in low‐moisture dry rooms, we expect that greater surface areas from engineered pores would increase degradation. In contrast, in situ crack formation would enable electrolyte penetration during cycling while minimizing side reactions during storage and electrode fabrication.

Based on recent research, we believe that intergranular cracking and electrolyte penetration contribute to decreased impedance in subsequent cycles compared to the first cycle, as often observed in this material. Other contributions include the removal of a passivating surface layer from atmospheric exposure of the cathode particles,^[^ ^124^, ^125^, ^126^ ^]^ or through autocatalytic effects when reaction and diffusion rates become faster with small amounts of delithiation.^[^ ^128^, ^129^ ^]^ Nonetheless, electrolyte penetration still contributes substantially to the reduced impedance through reducing the diffusion lengths and increasing the surface‐area‐to‐volume ratio that enables fast charge and discharge.

These findings show that intergranular cracking and electrolyte penetration have both positive and negative roles in polycrystalline NMC particles. The increased electrochemically‐active area results in faster degradation (Figure 2), but also enables faster charge and discharge in NMC cathodes by increasing the surface‐area‐to‐volume ratio and reducing the characteristic diffusion length.

## 5 Implications of Electrolyte Penetration Kinetics on Battery Modeling and Design

We consider the relative importance of electrolyte penetration in enabling fast charging in a number of battery systems. We will primarily discuss from the point of view of diffusion because it presents an intrinsic speed limit for fast charge and discharge and is less affected by interfaces.

### 5.1 Lithium Diffusion Coefficients

The lithium diffusion rate and the interfacial electrochemical reaction rate are two important parameters in understanding the kinetics of battery materials and for the accurate modeling and control of battery systems. Such parameters are usually obtained by combining a small‐signal electrochemical perturbation with separate measurements of the characteristic diffusion length and surface area of the particle. In earlier research, it was commonly assumed that the surface area and diffusion length are directly related to the diameter of the polycrystalline secondary particle.^[^ ^108^, ^110^, ^111^, ^130^, ^131^ ^]^ However, intergranular cracking and electrolyte penetration would substantially reduce the characteristic diffusion length while increasing the surface area. For this reason, measurements that do not account for cracking and electrolyte penetration would substantially overestimate the true lithium diffusion coefficient (D_Li_) and the exchange current density (j_0_), likely by more than an order of magnitude.^[^ ^120^ ^]^

In **Table** **1**, we review several reported measurements of the lithium diffusion coefficient, all obtained using small‐signal electrochemical techniques. Our summary shows an interesting trend: most reports that assume the diffusion length equals the size of the secondary particle report a diffusion coefficient on the order of 10^−10^ to 10^−9^ cm^2^ s^−1^. We believe these values overestimate the true lithium diffusivity because they assume a diffusion length on the order of 10 µm without accounting for electrolyte penetration.

**Table 1:** Reported values of lithium diffusion coefficients in NMC measured through electrochemical techniques. Most papers used polycrystalline particles without accounting for electrolyte penetration and thereby assumed the diffusion length equals the secondary particle radius. About 70% of these papers reported Li diffusivities between 0.8 × 10^−10^ and 1 × 10^‐^^9^ cm^2^s^−1^. In papers where electrolyte penetrations are accounted for or are not expected, all works reported much lower diffusion coefficients.

Reference	Composition	Li Diffusivity [cm^2^s^−1^]	Comment
*Polycrystalline NMC particles in liquid electrolyte without accounting for electrolyte penetration*			
Yang 2012^[^ ^132^ ^]^	NMC532	4.00E‐09	
Chaouchi 2021^[^ ^133^ ^]^	NMC622	2.00E‐09	
Charbonneau 2020^[^ ^134^ ^]^	NMC111	1.00E‐09	
Kang 2021^[^ ^135^ ^]^	NMC111	1.00E‐09	
Trevisanello 2021^[^ ^36^ ^]^	NMC811	1.00E‐09	Polycrystalline Particles, Liquid Electrolyte
Ruess 2020^[^ ^35^ ^]^	NMC811	1.00E‐09	Polycrystalline Particles, Liquid Electrolyte
Noh 2013^[^ ^62^ ^]^	NMC622	8.00E‐10	
Wang 2017^[^ ^136^ ^]^	NMC622	8.00E‐10	
Xu 2019^[^ ^137^ ^]^	NMC811	6.00E‐10	
Min 2023^[^ ^37^ ^]^	NMC532	5.00E‐10	Assume 5 µm radius (Secondary Particle)
Wen 2020^[^ ^131^ ^]^	NMC111	3.00E‐10	
Gunthur 2025^[^ ^111^ ^]^	NMC622	2.00E‐10	
Tsai 2018^[^ ^108^ ^]^	NMC111	1.50E‐10	
Chen 2022^[^ ^138^ ^]^	NMC622	1.00E‐10	
Zuo 2025^[^ ^120^ ^]^	NMC811	1.00E‐10	Assume 10 µm radius (Secondary Particle)
Wei 2015^[^ ^139^ ^]^	NMC622	8.00E‐11	
Cui 2016^[^ ^140^ ^]^	NMC622	8.00E‐11	
Ashton 2020^[^ ^141^ ^]^	NCA721	3.00E‐11	
Lakhdar 2023^[^ ^142^ ^]^	NMC622	2.00E‐11	
Verma 2017^[^ ^110^ ^]^	NMC532	2.00E‐11	
Nickol 2020^[^ ^143^ ^]^	NMC532	1.00E‐11	
*Polycrystalline NMC particles in liquid electrolyte that accounts for electrolyte penetration*			
Min 2023^[^ ^37^ ^]^	NMC532	1.00E‐11	Assume 500 nm radius
Zuo 2025^[^ ^120^ ^]^	NMC811	1.00E‐13	Assume 300 nm radius (Primary Particle)
*Electrolyte penetration is not expected*			
Ruess 2020^[^ ^35^ ^]^	NMC811	1.00E‐11	Solid Electrolyte
Trevisanello 2021^[^ ^36^ ^]^	NMC811	1.00E‐11	Single‐crystal Particle
Fan 2021^[^ ^144^ ^]^	NMC811	2.00E‐14	Single‐crystal Particle

A small number of papers do not assume the diffusion length equals the secondary particle size. Some experiments use single‐crystal NMC or solid electrolytes where electrolyte penetration is not expected;^[^ ^35^, ^36^, ^144^ ^]^ the reported diffusion coefficient is much lower, on the order of 10^−11^ to 10^−13^ cm^2^ s^−1^. Although the diffusion coefficient depends on the state of charge, our table only records the highest measured diffusion coefficient, typically obtained at a 30–50% delithiated particle, for ease of comparison. We note that a similar trend is expected for the exchange current density j_0_; however, such measurements usually show greater variation because they depend on the electrolyte and the interface and are more difficult to compare between reports.

Based on these findings, we believe that reporting traditional kinetic parameters of exchange current density (A m^−2^) and lithium diffusion coefficients (cm^2^ s^−1^) may be unsuitable for polycrystalline layered oxides because the surface area and diffusion length are not known. Instead, we propose that small‐signal electrochemical measurements should instead normalize the extracted kinetic parameters against variables that are not dependent on an assumed particle geometry. We propose that the exchange current normalized by the mass (A g^−1^) or normalized by the  capacity (A A^−1^ h^−1^) would be more appropriate; the latter also has units of inverse time. Similarly, the diffusion time (L^2^/4D_Li_) or its inverse D_Li_/L^2 [^ ^166^ ^]^ rather than the diffusion coefficient also provides a geometry‐agnostic method to compare solid diffusion rates. Because these values are not normalized against an assumed particle geometry, they provide a better comparison between different experiments, as well as for physical model development.

### 5.2 Simulation of Charging Times

**Figure 4:**
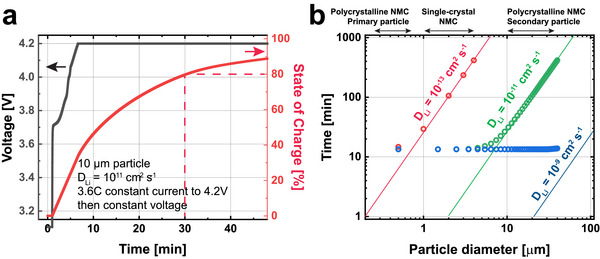
Simulations of fast charging based on different assumptions of particle diffusion length. a) PyBaMM simulation of fast charge taken under 3.6C constant current, followed by constant voltage for a monolithic solid particle. The time it takes to reach 80% SOC is recorded. b) Time to reach 80% SOC compared to a characteristic diffusion time of τ  = *L* ^2^ /4*D_Li_* where L equals ¼ of the diameter. This diffusion length is based on 7/8 of the volume in a spherical particle lies within ½ of the radius of the surface of the particle.

We simulate how these different assumptions for the diffusion coefficient control the time it takes to quickly charge and discharge a particle using PyBaMM simulations on NMC‐Graphite electrodes. We charge at constant current a rate of 3.6C, and then hold at constant voltage to 4.2V (details in Supporting Information; parameters from Marquis et al. 2019^[^ ^145^ ^]^). We assume a very fast interfacial reaction (j_0_ = 100 A m^−2^) to only consider the effects of diffusion coefficient and particle size because diffusion coefficients have been better quantified and are more intrinsic to the particles. We continue the use of exchange current density and lithium diffusion, despite their shortcomings, because the models assume these as input parameters. **Figure** **4**a shows an example of a simulated NMC particle under fast charging behavior with an assumed D_Li_ of 10^−11^ cm^2^s^−1^. The cell voltage reaches 4.2V quickly and takes ≈30 min to reach an 80% of the state‐of‐charge.

Figure 4b plots the simulated amount of time for fast charging as a function of the particle size for three diffusion coefficients: 10^−9^, 10^−11^, and 10^−13^ cm^2^s^−1^. As previously discussed in Table 1, we believe 10^−9^ cm^2^s^−1^ is too high because they do not account for electrolyte penetration. 10^−11^ cm^2^s^−1^ was the value obtained by Ruess et al. for solid electrolytes^[^ ^35^ ^]^ and Trevisanello et al. for single‐crystal NMC,^[^ ^36^ ^]^ both cases where electrolyte penetration is not expected (Figure 3a,d). This value was also obtained by Min et al., assuming an effective particle radius of 500 nm.^[^ ^37^ ^]^ A lithium diffusivity of 10^−13^ cm^2^s^−1^ was recently measured by Zuo et al. using single‐particle measurements for very Ni‐rich NMC under the assumption that the primary particle is the relevant length for diffusion.^[^ ^120^ ^]^ We compare these simulated times to the characteristic diffusion time (τ) obtained through $\tau =\frac{L^{2}}{4D_{Li}}\;$ where the *L* is the solid distance a lithium atom must diffuse from the electrolyte to the center of the particle. *L* is assumed to equal half the particle radius as 7/8 of the lithium lies within a distance L from the shell of a spherical particle.

Figure 4b shows our simulated time to reach 80% charge (circles) compared to the computed characteristic diffusion time (lines). The minimum charge time is ≈12 min, a result of the 3.6C constant current charge. When it exceeds this time, the simulated charge time is given by the characteristic diffusion time based on a $\tau =\frac{L^{2}}{4D_{Li}}\;$, where L equals ¼ the diameter. This confirms that the simulated particle is diffusion‐limited. This analysis shows that the characteristic diffusion time can be used to provide a very simple estimate for the fastest charge time of NMC cathodes.

Figure 4b has several important implications, which will be further discussed in subsequent sections. First, electrolyte penetration means that the diffusion time for a particle with an effective diameter of 1 µm is substantially shorter than 10 min, assuming D_Li_ = 10^−11^ cm^2^s^−1^. However, in the absence of electrolyte penetration, particles with a diameter larger than 10 µm will quickly become diffusion‐limited and not be able to undergo fast charge. According to the simulation where D_Li_ = 10^−11^ cm^2^s^−1^, 10‐µm particles can charge to 80% in ≈30 min, which is slower than the ≈15‐min mark for fast charging applications.^[^ ^146^, ^147^ ^]^ Interestingly, Park and coworkers recently reported a well‐engineered solid‐state battery that can charge and discharge at a rate of 2C^[^ ^106^ ^]^ where electrolyte penetration is not possible. We note that grain alignment may also somewhat improve the lithium diffusion coefficient.^[^ ^106^ ^]^

Our simplified analysis based on diffusion time can also be used to help understand when the negative role of intergranular cracks starts to outweigh the positives in terms of electrochemical kinetics, particularly due to the rise in cathode impedance. Assuming we use particles with a 10 µm diameter and a diffusion coefficient of 10^−11^ cm^2^s^−1^, the fastest rate that an NMC particle can be charged based on monolithic solid diffusion without electrolyte penetration is ≈30 min. In contrast, there are many examples of fast charging applications at 6C and 9C which show more than 500^[^ ^148^, ^149^ ^]^ or even 1000^[^ ^150^ ^]^ cycles, including at low temperatures. This analysis shows that electrolyte penetration offers benefits for electrochemical kinetics despite the increased degradation even after many charge/discharge cycles, compared to a hypothetical monolithic particle which does not crack.

### 5.3 Single‐Crystal NMC

In polycrystalline NMC, intergranular cracks between primary particles provide pathways for electrolyte infiltration, increasing electrochemical surface area and accelerating ion transport. Single‐crystalline NMC lacks this secondary particle structure; as a result, intergranular cracks are absent, and the electrolyte cannot penetrate the particle, thereby reducing degradation. Although intragranular cracks may form at high cutoff voltages (> 4.5 V) due to the stacking sequence phase transition,^[^ ^71^ ^]^ they do not appear to substantially increase degradation under lower‐voltage cycling conditions (< 4.3 V). Based on this assessment, single‐crystal NMC shows improved capacity retention compared to polycrystalline ones.^[^ ^8^, ^30^, ^32^, ^104^, ^105^, ^151^, ^152^ ^]^ This is especially true when the single‐crystal NMCs are larger.

While single‐crystal NMC usually shows improved capacity retention,^[^ ^8^, ^105^, ^151^ ^]^ the evidence for the rate kinetics has been mixed. In porous electrodes, different single‐crystal materials have shown improved,^[^ ^105^, ^153^, ^154^ ^]^ similar,^[^ ^155^ ^]^ or worse^[^ ^27^, ^58^ ^]^ rate capability compared to polycrystalline counterparts. However, a closer examination of the reported studies reveals that the cases with superior single‐crystal performance often involved commercial polycrystalline materials as the control group^[^ ^105^, ^153^, ^155^ ^]^ or employed different techniques for synthesizing single‐crystal particles.^[^ ^154^ ^]^ In studies where variables were strictly controlled through in‐house synthesis, single‐crystal materials exhibited lower rate capability than polycrystalline ones.^[^ ^27^, ^58^ ^]^ Among the synthesis parameters, calcination temperature and time are the most influential factors affecting single‐crystal formation. When these parameters were controlled to enable direct comparison between single‐ and polycrystalline materials, single‐crystal particles showed poorer rate performance.

In porous electrodes, an additional kinetic shortcoming of single‐crystal NMC is the inferior electronic contact between the smaller single‐crystal particle and the electronically conductive carbon black network,^[^ ^156^ ^]^ which is also observed in single‐crystal LiFePO_4_ nanoparticles.^[^ ^157^ ^]^ Oh, and coworkers showed that this shortcoming can be substantially mitigated by mixing larger polycrystalline and smaller single‐crystal particles.^[^ ^156^ ^]^ This poor electronic contact could explain why single‐crystal NMC would struggle at high rates, even though the characteristic diffusion time can be as short as 1 or 2 min (Figure 4b). For this reason, while moderate reductions in particle size (e.g., from 5 to 2 µm) would yield faster particles due to shorter diffusion lengths (Figure 4) and larger surface areas, additional reduction to submicron dimensions may not ultimately yield faster cycling because it could worsen the electronic connectivity of the particles without introducing large amounts of conductive additives. Moreover, smaller particles would also increase the surface area, which could increase surface degradation.

While NMC particles benefit from electrolyte penetration for fast cycling rates, micron‐sized single‐crystal LiCoO_2_ particles show no such requirement. Recent work by Suk and colleagues showed robust electrochemical cycling at 20C and higher on a >10 µm LiCoO_2_ single‐crystal particle.^[^ ^50^ ^]^ This result suggests that LiCoO_2_ particles have much faster lithium diffusion and interfacial reaction kinetics than NMC particles. In contrast, 2 µm NMC particles struggle at rates of 5C.^[^ ^38^ ^]^

### 5.4 Solid‐State Batteries

Solid‐state batteries may offer high energy densities and improved safety compared to conventional lithium‐ion systems.^[^ ^158^, ^159^, ^160^ ^]^ Although polycrystalline particles are commonly used in Li‐ion batteries in part due to fast charging rates enabled by electrolyte penetration, such particles would not benefit from these cracks in solid‐state batteries.^[^ ^161^ ^]^ Ruess and colleagues show that the apparent lithium diffusion coefficient in solid electrolytes is ≈100 times lower than in liquid electrolytes using the same polycrystalline NMC particles due to the lack of electrolyte penetration in solid electrolytes (Figure 3a).^[^ ^35^ ^]^ Based on our analysis in Figure 4, we predict that 2C is the fastest that a 10‐µm polycrystalline NMC can charge in the absence of liquid electrolyte penetration. This rate was recently achieved by Park et al. through doping, grain alignment, and interfacial engineering.^[^ ^106^ ^]^

Given that cracks are not expected to benefit the kinetics of all‐solid‐state batteries, single‐crystal cathodes may be preferred over polycrystalline ones, assuming the electronic contact issues can be resolved.^[^ ^156^ ^]^ Single‐crystal LiCoO_2_, which has much faster lithium diffusion without the need for electrolyte penetration, may be preferred for certain solid‐state battery applications^[^ ^162^, ^163^, ^164^ ^]^ that require fast cycling. Alternatively, the addition of a liquid catholyte for hybrid batteries^[^ ^165^ ^]^ may also facilitate improved kinetics if polycrystalline NMC or NCA particles are desired.

## 6 Conclusion

In this Perspective, we propose that intergranular cracking in polycrystalline layered oxide cathodes, commonly synthesized via co‐precipitation, not only degrades the cathode but also improves the electrochemical kinetics. Lattice contraction and expansion during cycling induce stress between primary particles, leading to the formation of cracks. These cracks expand the reaction surface area and have traditionally been interpreted solely as a cause of accelerated capacity loss. However, the potential benefits of surface area enhancement have often been largely overlooked, primarily because capacity degradation is easy to quantify, while the electrochemical surface area that underpins kinetic analysis is much more difficult to measure accurately.

In conclusion, intergranular cracking in polycrystalline layered oxide particles represents a double‐edged sword. While it contributes to capacity fade, it also serves as a structural feature that enables fast electrochemical kinetics, which is a key performance metric for lithium‐ion batteries. Recognizing the dual nature of cracking not only offers a new tool for interpreting irreversible early‐cycle behaviors but also opens new pathways for cathode material design. For future studies, approaches should go beyond simply suppressing or avoiding cracks and instead focus on actively leveraging and optimizing them through intentional design.

## Conflict of Interest

The authors declare no conflict of interest.

## Supporting information



Supporting Information
