# Supplementary material for: The Role of Intergranular Cracks on Fast Charging and Accelerated Degradation in Polycrystalline Layered Oxide Cathodes
Source: Adv Sci (Weinh). 2025 Oct 29;13(3):e15588. doi: 10.1002/advs.202515588 (PMC12806215; doi:10.1002/advs.202515588)
Supplement: Supplementary file 1 — Supporting Information [file ADVS-13-e15588-s001.pdf]

Supporting Information for

The Role of Intergranular Cracks on Fast Charging and Accelerated Degradation in Polycrystalline Layered Oxide Cathodes

Jinhong Min, Tobias Glossmann, Yiyang Li

Lithium-ion Cell Simulations (PyBaMM)

Electrochemical model and parameters

All simulations were performed in Python (version 3.12.7) using PyBaMM (version 25.6.0). We used PyBaMM's lithium-ion Single Particle Model (SPM) with the Marquis2019 parameter set. Unless otherwise stated, parameters follow the set defaults. For state-of-charge (SOC) post-processing, we used a nominal cell capacity of 0.95 Ah to convert charge capacity to SOC.

Variable	Unit	Value
Ambient temperature	K	298.15
Boltzmann constant	$J.K^{-1}$	1.38065E-23
Bulk solvent concentration	$mol.m^{-3}$	2636
Cation transference number	-	0.4
Electrolyte conductivity	$S.m^{-1}$	function, Capiglia1999
Electrolyte diffusivity	$m^2.s^{-1}$	function, Capiglia1999
Electron charge	C	1.60218E-19
Faraday constant	$C.mol^{-1}$	96485.33212
Ideal gas constant	$J.K^{-1}.mol^{-1}$	8.314462618
Initial concentration in electrolyte	$mol.m^{-3}$	1000
Initial concentration in negative electrode	$mol.m^{-3}$	19986.6096
Initial concentration in positive electrode	$mol.m^{-3}$	30730.75544
Negative electrode charge transfer coefficient	-	0.5
Negative electrode conductivity	$S.m^{-1}$	100
Negative electrode double-layer capacity	$F.m^{-2}$	0.2
Negative electrode exchange-current density	$A.m^{-2}$	function, graphite Dualfoil1998
Negative electrode porosity	-	0.3
Negative electrode thickness	m	0.0001
Negative particle diffusivity	$m^2.s^{-1}$	function, graphite Dualfoil1998
Negative particle radius	m	0.00001
Positive electrode charge transfer coefficient	-	0.5
Positive electrode conductivity	$S.m^{-1}$	10

Positive electrode double-layer capacity	$F.m^{-2}$	0.2
Positive electrode exchange-current density	$A.m^{-2}$	function, LiCoO2 Dualfoil1998
Positive electrode porosity	-	0.3
Positive electrode thickness	M	0.0001
Positive particle diffusivity	$m2.s^{-1}$	function, LiCoO2 Dualfoil1998
Positive particle radius	m	0.00001

Table S1. List of important default variables among the parameters used in the PyBaMM model.

Charging protocol (experiment definition)

The charging experiment was specified through PyBaMM

1. Rest for 1 min
2. Constant-current charge at 3.6 C until 4.20 V
3. Constant-voltage hold at 4.20 V until C/20 cutoff

For the parameter-sweep study, an additional post-charge rest of 0.1 h was appended. The initial SOC was set to 0%.

Representative charging simulation

To examine a representative charge, we modified three positive-electrode properties

Positive particle diameter: 5 μm

Positive-electrode exchange-current density: 100 A m^{-2}

Positive-electrode solid-phase Li diffusivity: 1.0×10^{-11} cm^2 s^{-1}

Radius sweep simulation

To study diffusion limitations, we swept the positive-particle diameter from 0.5 to 40 μm and repeated the charge experiment for three solid diffusivities in the positive electrode:

Positive particle diameter: 0.5 to 40 μm

Positive-electrode exchange-current density: 100 A m^{-2}

Positive-electrode solid-phase Li diffusivity: 1.0×10^{-9}, 1.0×10^{-11}, 1.0×10^{-13} cm^2 s^{-1}
